# The diagnostic value of admission myoglobin for sepsis-induced cardiomyopathy: a prospective cohort study

**DOI:** 10.3389/fcvm.2026.1921535

**Published:** 2026-10-12

**Authors:** Yue Chen, Xiaohong Lin, Guangwei Yu

**Affiliations:** 1 Department of Emergency, Fujian Medical University Union Hospital, Fuzhou, Fujian, China; 2 Fujian Key Laboratory of Vascular Aging, Fujian Medical University, Fuzhou, China

**Keywords:** early diagnosis, myoglobin, NT-proBNP, ROC curve, sepsis-induced cardiomyopathy, SOFA score

## Abstract

**Background:**

Sepsis-induced cardiomyopathy (SICM) is a frequent and under-recognized complication of sepsis. Early diagnosis remains challenging. We evaluated the diagnostic value of admission myoglobin (MYO), alone and combined with NT-proBNP and the SOFA score, for SICM in patients with sepsis.

**Methods:**

In this single-center prospective cohort study (April 2022 to May 2024), we enrolled 158 patients with sepsis diagnosed according to Sepsis-3 criteria. Blood samples for MYO and NT-proBNP were collected within 24 h of admission; admission echocardiography was performed as soon as feasible (within 24 h whenever possible, and within 72 h in all enrolled patients); follow-up echocardiography was performed between day 7 and day 14. SICM was defined as left ventricular ejection fraction (LVEF) below 50% with documented reversibility. Univariate and multivariate logistic regression analyses were performed to identify factors associated with SICM, adjusting for age, sex, body mass index, and renal function. Receiver operating characteristic (ROC) curve analysis, internal validation by 1,000 bootstrap resamples, and decision curve analysis (DCA) were performed. Model calibration was assessed by a calibration plot, the calibration intercept and slope, and the Brier score, with bootstrap optimism-corrected estimates.

**Results:**

Of 158 patients, 63 (39.9%) were diagnosed with SICM. MYO levels were significantly higher in the SICM group than in the non-SICM group (median 395.7 vs. 97.3 ng/mL, *P* < 0.001). In multivariate analysis adjusting for age, sex, BMI, and renal function, MYO (OR 1.061 per 100 ng/mL, 95% CI 1.008–1.115; *P* = 0.023) and NT-proBNP (OR 1.087 per 1,000 pg/mL, 95% CI 1.025–1.153; *P* = 0.005) were independently associated with SICM. The combined model (MYO + NT-proBNP + SOFA) achieved an area under the ROC curve (AUC) of 0.821 (95% CI: 0.750–0.892), outperforming SOFA alone (AUC 0.743; likelihood ratio test *P* < 0.001); the optimism-corrected AUC after bootstrap validation was 0.812. The model was well calibrated (calibration intercept 0.00, slope 1.00; Brier score 0.173). DCA suggested potential net benefit across threshold probabilities of 0.1–0.7.

**Conclusion:**

Admission MYO, particularly in combination with NT-proBNP and SOFA, may assist in the early identification and diagnosis of SICM.

## Introduction

Sepsis is a life-threatening organ dysfunction clinical syndrome caused by the body's dysregulated response to infection, with a high incidence and mortality rate, and poses a serious threat to human life and health worldwide. In the Intensive Care Unit (ICU), sepsis is also a major cause of death among critically ill patients (1). SICM is a severe complication in sepsis that significantly affects patient prognosis, but there is still uncertainty about the exact definition of SICM. Different studies have used different definitions and criteria, leading to differences in understanding the epidemiology, clinical significance, and treatment strategies for SICM. In clinical application, we need clearer biomarkers to identify patients with SICM (2, 3). MYO is a small molecular oxygen-binding protein found in cardiac and skeletal muscle, and its main function is to promote the diffusion and storage of oxygen in muscle tissue. Yao et al. found in their 2016 study that MYO levels can be detected in the early stages of sepsis and may serve as a potential biomarker for assessing the severity and prognosis of sepsis (4).

In this study, SICM was defined according to established echocardiographic criteria as left ventricular ejection fraction (LVEF) below 50% with documented reversibility on follow-up imaging (5, 6). We hypothesized that MYO, an early and sensitive marker of muscle injury, may serve as a clinically useful biomarker in patients with SICM. The primary aim of this study was to determine the diagnostic value of admission MYO for SICM; the secondary aim was to evaluate whether combining MYO with NT-proBNP and the SOFA score improves diagnostic accuracy compared with SOFA alone.

## Methods

### Study design and population

This single-center prospective cohort study was conducted at the emergency department of Fujian Medical University Union Hospital between April 2022 and May 2024. The study protocol was approved by the Research Ethics Committee of Fujian Medical University Union Hospital (Ethics Code: 2022YF004-02) and registered with the Chinese Clinical Trial Registry (ChiCTR2200058007). Written informed consent was obtained from all participants or their legal representatives.

A total of 287 patients were screened; 158 patients were enrolled in the final analysis (Figure 1). The inclusion criteria were: (1) age 18 years or older; and (2) meeting the Sepsis-3 diagnostic criteria (suspected or confirmed infection with an acute increase of at least 2 points in the SOFA score) (1). The exclusion criteria were: (1) persistent or permanent atrial fibrillation or other severe arrhythmias; (2) pregnancy; (3) congenital heart disease; (4) moderate-to-severe valvular disease or prior valve replacement surgery; (5) history of acute myocardial infarction, myocarditis, or chronic heart failure; (6) end-stage malignancy; (7) diagnosis of sepsis in another ward followed by transfer more than 24 h after onset; and (8) concurrent participation in other studies. Patients who died within 72 h of admission before the scheduled echocardiographic examination could be performed—despite resuscitation efforts and attempted blood sampling on arrival—were excluded because SICM status could not be ascertained.

**Figure 1:**
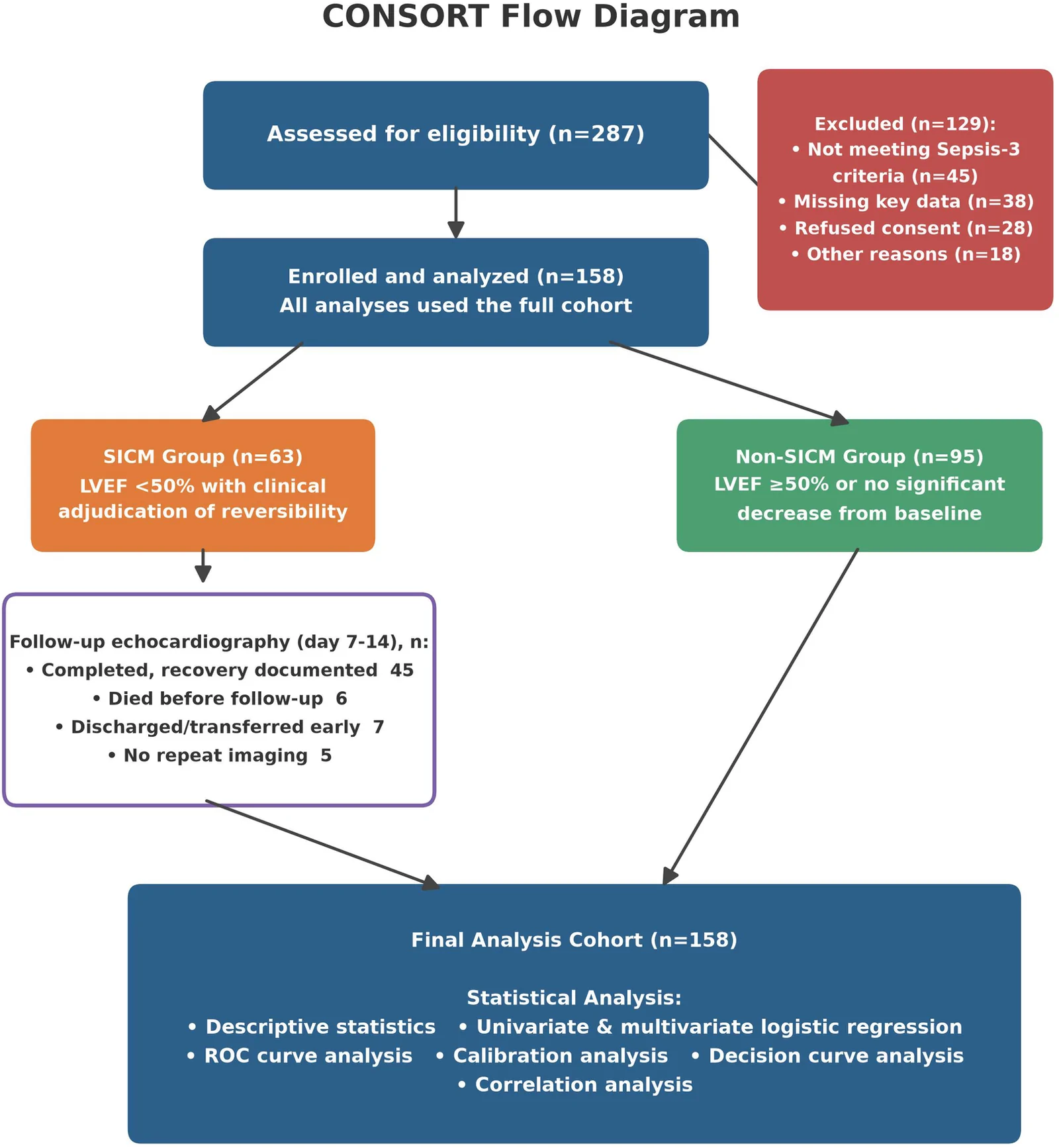
CONSORT flow diagram of patient enrollment. Of 287 patients assessed for eligibility, 158 were enrolled and divided into SICM (*n* = 63) and non-SICM (*n* = 95) groups. Among the 63 patients with SICM, 45 completed follow-up echocardiography between days 7 and 14 with documented recovery, six died before follow-up, seven were discharged or transferred early, and five had no repeat imaging.

### Data collection

Demographic data (age, sex, body mass index) and admission laboratory variables were collected within 24 h of emergency department presentation, prior to any study-related intervention. Laboratory measurements included white blood cell count, neutrophil-to-lymphocyte ratio, platelet count, C-reactive protein, procalcitonin, lactate, alanine aminotransferase, aspartate aminotransferase, estimated glomerular filtration rate (eGFR), creatinine, glucose, lactate dehydrogenase, creatine kinase MB, troponin I, interleukin-6, MYO, and NT-proBNP. The SOFA score was calculated from the worst physiological variables within the first 24 h. All laboratory assays were performed by the central clinical laboratory of our institution using standardized methods. Serum MYO was measured by an immunoturbidimetric assay and NT-proBNP by an electrochemiluminescence immunoassay, both performed on automated analyzers in the central clinical laboratory of our institution using commercial reagents according to the manufacturers’ instructions; the manufacturers’ reference intervals were applied (MYO 25–58 ng/mL; NT-proBNP 0–349 pg/mL). Blood samples were collected in serum separator tubes, allowed to clot at room temperature, centrifuged, and analyzed on the day of collection. Samples with visible hemolysis or lipemia were re-collected when feasible; otherwise biomarker results were interpreted with caution. Assay precision met the manufacturers’ specifications, with routine internal quality control performed daily in the central laboratory.

### Echocardiography and definition of SICM

All participants underwent transthoracic echocardiography (TTE) as soon as feasible after admission (within 24 h whenever possible, and within 72 h in all enrolled patients). Each examination was performed and interpreted by a single reader from a team of six experienced cardiologists (attending or senior physician level), who were blinded to biomarker results and to all clinical data, including the working diagnosis. Left ventricular ejection fraction (LVEF) was quantified by the modified biplane Simpson method in accordance with international echocardiography guidelines. Standardized image-acquisition protocols were used, and measurements were repeated after image optimization whenever the acoustic window or the automated estimate was judged inadequate. Follow-up echocardiography was performed between day 7 and day 14 after admission, using the same method and blinding procedures, to assess the reversibility of ventricular dysfunction. The median time from emergency department arrival to blood sampling was 1 h (approximately 60 min), and the median time to echocardiography was 2.5 h (approximately 150 min); in all enrolled patients both were completed within 72 h of admission.

SICM was defined as LVEF below 50% together with documented reversibility, consistent with established criteria for sepsis-associated reversible myocardial depression (5, 6). Baseline LVEF (from imaging performed within 6 months before admission) was used when available to confirm a decline of at least 10 percentage points. Because baseline LVEF was not systematically documented in all patients, the operational diagnosis in this cohort relied on admission LVEF below 50% with clinical adjudication of reversibility on follow-up echocardiography (day 7–14). The 10% change refers to absolute percentage points, not a relative change. Patients who died before follow-up imaging were retained in the SICM group on the basis of their admission findings, but they were not counted as having documented reversibility; their outcomes are reported transparently in the Results section.

### Statistical analysis

Data were analyzed using SPSS 26.0 (IBM Corp., Armonk, NY, USA). Continuous variables with normal distribution were expressed as mean +/- standard deviation and compared by independent-sample t-tests; non-normally distributed variables were expressed as medians (interquartile range, IQR) and compared by Mann–Whitney U tests. Categorical variables were compared by chi-square tests. All analyses—descriptive comparisons, logistic regression, ROC analysis, calibration analysis, decision curve analysis, and correlation analysis—were conducted in the full analytic cohort (*n* = 158). There were no missing data for any variable included in the regression models or the combined diagnostic model; every analysis therefore included all 158 patients (complete-case analysis; no imputation was performed).

Univariate logistic regression was performed for each candidate predictor. Based on the univariate results and clinical relevance, SOFA score, platelet count, MYO, NT-proBNP, and PCT were entered as candidate predictors into the multivariable adjusted association model (Table 3), together with the prespecified covariates age, sex, BMI, and eGFR (renal function); TnI, lactate, and septic shock were not entered to limit collinearity with the primary biomarkers and to maintain an adequate events-per-variable ratio. Second, a parsimonious diagnostic model comprising only MYO, NT-proBNP, and the SOFA score was fitted and used for ROC analysis, decision curve analysis, and calibration. The coefficients of MYO and SOFA in this diagnostic model differ from those in the adjusted association model because the latter additionally adjusts for demographic covariates, renal function, and competing laboratory predictors; both models are reported in full (Table 3 and Table 5). The association model was additionally adjusted for CK-MB in a sensitivity analysis. Information on trauma, rhabdomyolysis, and recent surgery was not systematically recorded in this cohort and could not be analyzed. Odds ratios (ORs) for MYO and NT-proBNP are reported on clinically interpretable scales (per 100 ng/mL and per 1,000 pg/mL, respectively), with the unscaled coefficients given in Table 3 so that the equation can be reproduced; the OR for MYO is additionally expressed per 1-standard-deviation (SD) increase. Model discrimination was assessed by receiver operating characteristic (ROC) curve analysis with the Youden index used to identify optimal cutoffs. Because these cutoffs were selected and evaluated in the same cohort (n = 158) without an independent validation set, they should be regarded as exploratory rather than as validated clinical thresholds. To evaluate the incremental value of biomarkers, the combined model (MYO + NT-proBNP + SOFA) was compared against SOFA alone and against intermediate models using likelihood ratio tests. Internal validation was performed with 1,000 bootstrap resamples, repeating the entire model-building procedure in each resample, and the optimism-corrected area under the curve (AUC) is reported. Calibration of the combined model was assessed with a calibration plot (ten quantile groups), the calibration intercept and slope (from a logistic regression of the outcome on the logit of the predicted probability), and the Brier score; bootstrap optimism-corrected calibration measures were obtained. Decision curve analysis was used to estimate the potential net benefit of the combined model across a range of threshold probabilities. Spearman rank correlation was used to explore associations among biomarkers and clinical variables. Two-sided *P* < 0.05 was considered statistically significant.

## Results

### Patient enrollment and baseline characteristics

Of 287 screened patients, 158 were enrolled and included in the analysis; 63 (39.9%) were diagnosed with SICM and 95 (60.1%) had no SICM (Figure 1). The median age was 64 years (IQR 54–75), and 94 patients (59.5%) were male. Baseline characteristics are shown in Table 1. Compared with non-SICM patients, patients with SICM were older (68 +/- 14 vs. 62 +/- 15 years, *P* = 0.007) and had lower platelet counts (*P* = 0.002), lower eGFR (*P* = 0.011), and higher SOFA scores (*P* < 0.001). All variables in Table 1 were measured at admission (within 24 h).

**Table 1:** Clinical characteristics of the 158 sepsis patients (full cohort).

Variable	SICM (*n* = 63)	non-SICM (*n* = 95)	*P*
Male/Female	38/25	56/39	0.864
Age, years	68 ± 14	62 ± 15	0.007
BMI, kg/m2	24.5 ± 3.9	23.5 ± 3.8	0.102
WBC, ×10^9^/L	13.4 (7.9–19.7)	12.2 (7.3–24.1)	0.798
PLT, ×10^9^/L	106.0 (32.0–173.0)	166.5 (82.0–255.5)	0.002
NLR	15.4 (7.0–31.0)	15.5 (9.4–33.9)	0.461
CRP, mg/L	127.7 (54.9–195.8)	141.8 (66.6–218.3)	0.324
PCT, ng/mL	22.9 (5.3–100.0)	9.9 (1.4–38.4)	0.051
Lactate, mg/dL	39.4 (22.1–61.5)	30.8 (19.2–44.4)	0.022
ALT, IU/L	40.0 (18.0–68.0)	34.0 (21.5–64.3)	0.401
AST, IU/L	63.0 (31.0–190.0)	45.0 (29.5–83.8)	0.031
eGFR, mL/min/1.73m2	36.4 (23.0–64.9)	56.5 (34.2–101.8)	0.011
MYO, ng/mL	395.7 (131.2–1486.0)	97.3 (58.6–330.0)	<0.001
NT-proBNP, pg/mL	3635 (1158–13765)	944 (325–3180)	<0.001
TnI, ug/L	0.100 (0.027–0.342)	0.006 (0.004–0.018)	<0.001
LDH, IU/L	346.0 (238.7–572.0)	298.5 (234.8–457.0)	0.229
IL-6, pg/mL	637.4 (74.7–3061.0)	322.0 (96.4–966.7)	0.292
LVEF, %	40 (37–44)	61 (57–67)	<0.001
SOFA score	8 (6–10)	5 (3–7)	<0.001

Values are median (IQR), mean +/- SD, or n/n. All variables were measured at admission (within 24 h of emergency department presentation), prior to any study-related intervention. MYO, myoglobin; NT-proBNP, N-terminal pro-B-type natriuretic peptide; TnI, troponin I; LVEF, left ventricular ejection fraction; SOFA, Sequential Organ Failure Assessment; WBC, white blood cell count; PLT, platelet; NLR, neutrophil-to-lymphocyte ratio; CRP, C-reactive protein; PCT, procalcitonin; eGFR, estimated glomerular filtration rate; LDH, lactate dehydrogenase; IL-6, interleukin-6; BMI, body mass index.

Of the 63 patients with SICM, 45 (71.4%) completed follow-up echocardiography between days 7 and 14, and all 45 demonstrated recovery—an increase in LVEF to at least 50% or by at least 10 percentage points from admission—documenting the reversible nature of the ventricular dysfunction. Six patients died before follow-up imaging, seven were discharged or transferred before day 7, and five had no repeat imaging (three because of clinical improvement and two because of ongoing hemodynamic instability). These 18 patients were retained in the SICM group on the basis of admission LVEF below 50% and were not counted as having documented reversibility (Figure 1).

### Biomarker levels

Patients with SICM had significantly higher MYO (median 395.7 vs. 97.3 ng/mL, *P* < 0.001), NT-proBNP (median 3635 vs. 944 pg/mL, *P* < 0.001), troponin I (*P* < 0.001), procalcitonin (*P* = 0.051), and lactate (*P* = 0.022) levels than patients without SICM (Table 1).

### Univariate analysis

In univariate logistic regression, age, SOFA score, MYO, NT-proBNP, procalcitonin, troponin I, lactate, and septic shock were significantly associated with SICM, whereas sex, BMI, and eGFR were not (Table 2).

**Table 2:** Univariate logistic regression analysis of factors associated with SICM (*n* = 158).

Variable	OR	95% CI	*P*
Age (per year)	1.033	1.009–1.059	0.008
Male sex	1.059	0.553–2.027	0.864
BMI (per kg/m2)	1.072	0.986–1.166	0.103
SOFA (per point)	1.295	1.160–1.445	<0.001
PLT (per 10^9^/L)	0.996	0.992–0.999	0.006
MYO (per 100 ng/mL)	1.101	1.049–1.157	<0.001
NT-proBNP (per 1,000 pg/mL)	1.128	1.057–1.204	<0.001
PCT (per ng/mL)	1.010	1.001–1.019	0.025
TnI (per ug/L)	6.743	1.589–28.613	0.010
eGFR (per mL/min/1.73m2)	0.997	0.992–1.002	0.186
Lactate (per mg/dL)	1.015	1.003–1.027	0.014
Septic shock (yes vs. no)	3.28	1.67–6.45	<0.001

OR, odds ratio; CI, confidence interval; LVEF is not included because it forms part of the SICM case definition. Septic shock was coded No = 0, Yes = 1. MYO is reported per 100 ng/mL and NT-proBNP per 1,000 pg/mL.

### Multivariate analysis

In multivariate logistic regression (model specification in Table 3: prespecified covariates age, sex, BMI, and eGFR, plus candidate predictors SOFA score, platelet count, MYO, NT-proBNP, and PCT), MYO (OR 1.061 per 100 ng/mL, 95% CI 1.008–1.115, *P* = 0.023), NT-proBNP (OR 1.087 per 1,000 pg/mL, 95% CI 1.025–1.153, *P* = 0.005), and SOFA score (OR 1.175 per point, 95% CI 1.028–1.342, *P* = 0.018) remained independently associated with SICM; eGFR was not independently associated (*P* = 0.663) (Table 3).

**Table 3:** Multivariate logistic regression analysis of factors associated with SICM (*n* = 158).

Variable	B	SE	Wald	*P*	OR	95% CI
Age (per year)	0.018	0.015	1.50	0.221	1.018	0.989–1.047
Male sex	0.019	0.430	0.00	0.965	1.019	0.439–2.367
BMI (per kg/m2)	0.092	0.053	3.02	0.082	1.096	0.988–1.216
SOFA (per point)	0.161	0.068	5.63	0.018	1.175	1.028–1.342
PLT (per 10^9^/L)	−0.001	0.002	0.24	0.624	0.999	0.995–1.003
MYO (per 100 ng/mL)	0.059	0.026	5.21	0.023	1.061	1.008–1.115
NT-proBNP (per 1,000 pg/mL)	0.084	0.030	7.82	0.005	1.087	1.025–1.153
PCT (per ng/mL)	0.003	0.006	0.22	0.636	1.003	0.992–1.014
eGFR (per mL/min/1.73m2)	−0.001	0.002	0.19	0.663	0.999	0.995–1.003

The model includes the prespecified covariates age, sex, BMI, and eGFR (renal function), together with the candidate predictors SOFA score, platelet count, MYO, NT-proBNP, and PCT (*n* = 158, complete cases). MYO is reported per 100 ng/mL and NT-proBNP per 1,000 pg/mL; the corresponding unscaled coefficients are B = 0.0006 (SE 0.0003) per 1 ng/mL for MYO and B = 0.00008 (SE 0.00003) per 1 pg/mL for NT-proBNP, allowing the full equation to be reproduced. For clinical interpretability, the OR for MYO corresponds to 1.63 per 1-SD increase (SD 832 ng/mL) and 1.32 per interquartile range (473 ng/mL).

For clinical interpretability, the OR for MYO corresponds to 1.06 per 100 ng/mL increase and 1.63 per 1-SD increase (SD 832 ng/mL). In this cohort, the interquartile range of MYO spanned 473 ng/mL, corresponding to an OR of 1.32 across the IQR. For NT-proBNP, the OR corresponds to 1.09 per 1,000 pg/mL increase (95% CI 1.03–1.15).

In a sensitivity analysis with additional adjustment for CK-MB, the association between MYO and SICM was attenuated and was no longer statistically significant (*P* = 0.294), whereas CK-MB was independently associated with SICM (OR 1.02 per 1 U/L, 95% CI 1.01–1.04, *P* = 0.009); MYO and CK-MB were moderately correlated (rho = 0.572), indicating that part of the MYO signal may reflect skeletal muscle injury.

### Discrimination and internal validation

The AUCs for identifying SICM were 0.704 (95% CI 0.619–0.789) for MYO, 0.731 (95% CI 0.649–0.813) for NT-proBNP, and 0.743 (95% CI 0.662–0.824) for the SOFA score (Table 4; Figure 2). The combined model (MYO + NT-proBNP + SOFA) achieved an AUC of 0.821 (95% CI 0.750–0.892), with a sensitivity of 0.714, specificity of 0.779, and an optimal probability cutoff of 0.383.

**Table 4:** Diagnostic performance of MYO, NT-proBNP, SOFA, and the combined model for SICM (*n* = 158).

Predictor	AUC (95% CI)	Cutoff	Sensitivity	Specificity	*P*
MYO	0.704 (0.619–0.789)	156.7 ng/mL	0.714	0.663	<0.001
NT-proBNP	0.731 (0.649–0.813)	1,630 pg/mL	0.714	0.653	<0.001
SOFA	0.743 (0.662–0.824)	6	0.762	0.642	<0.001
MYO + NT-proBNP + SOFA	0.821 (0.750–0.892)	0.383	0.714	0.779	<0.001

The combined model (MYO + NT-proBNP + SOFA) significantly outperformed SOFA alone (likelihood ratio chi-square = 21.91, *P* < 0.001). Addition of MYO alone to SOFA: AUC 0.779, chi-square = 8.35, *P* = 0.002. Addition of NT-proBNP alone to SOFA: AUC 0.794, chi-square = 13.52, *P* < 0.001. Bootstrap internal validation (1,000 resamples): optimism-corrected AUC 0.812. Combined model equation: logit(P) = −2.428 + 0.172  ×  SOFA + 0.0007  ×  MYO + 0.0001  ×  NT-proBNP. Calibration of the combined model: intercept 0.00, slope 1.00, Brier score 0.173; bootstrap optimism-corrected values −0.04, 0.92, and 0.183. Full coefficients of the combined model are given in Table 5. Cutoffs were derived and evaluated in the same cohort and are exploratory; external validation is required before clinical application.

**Figure 2:**
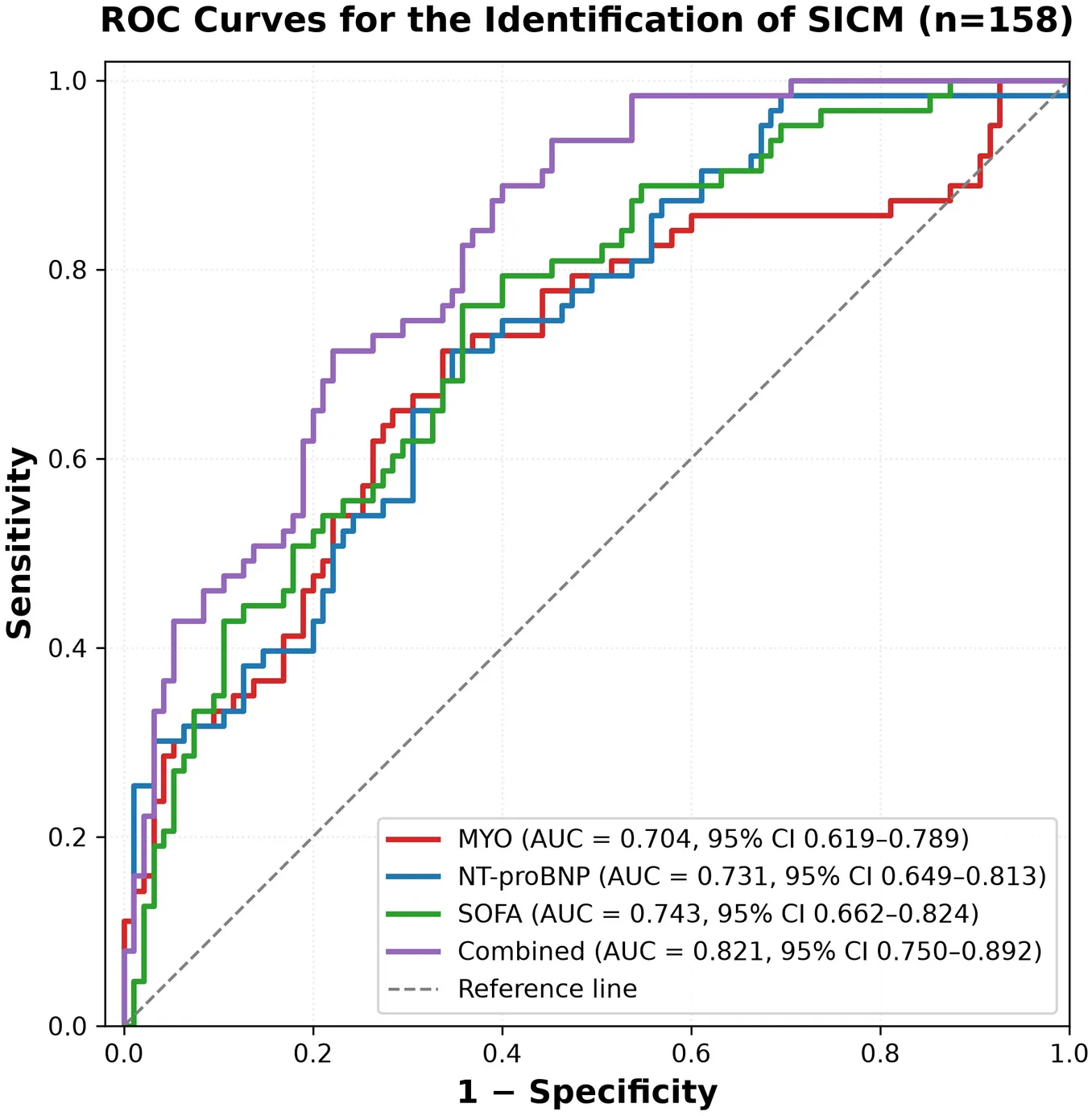
Receiver operating characteristic (ROC) curves for the identification of SICM in the full cohort (*n* = 158). Shown are MYO (AUC 0.704, 95% CI 0.619−0.789), NT-proBNP (AUC 0.731, 95% CI 0.649-0.813), SOFA (AUC 0.743, 95% CI 0.662−0.824), and the combined model MYO + NT-proBNP + SOFA (AUC 0.821, 95% CI 0.750−0.892; cutoff 0.383, sensitivity 0.714, specificity 0.779). The combined model significantly outperformed SOFA alone (*P* < 0.001).

The combined model significantly outperformed SOFA alone (AUC 0.821 vs. 0.743; likelihood ratio chi-square = 21.91, *P* < 0.001). Addition of MYO alone to SOFA significantly improved discrimination (AUC 0.779 vs. 0.743; chi-square = 8.35, *P* = 0.002), as did addition of NT-proBNP alone (AUC 0.794; chi-square = 13.52, *P* < 0.001). The incremental contribution of MYO beyond SOFA and NT-proBNP was further quantified by an integrated discrimination improvement (IDI) of 0.041 (95% CI 0.001–0.134) and a category-free net reclassification improvement (NRI) of 0.383 (95% CI −0.030–0.806).

In 1,000 bootstrap resamples, the optimism-corrected AUC of the combined model was 0.812 (apparent AUC 0.821), indicating minimal overfitting. The fitted combined model is: logit(P) = −2.428 + 0.172  ×  SOFA + 0.0007  ×  MYO + 0.0001  ×  NT-proBNP, where MYO is in ng/mL and NT-proBNP is in pg/mL. No predictor transformations were applied, and there were no missing data in the model covariates. Calibration was good: the calibration intercept was 0.00, the calibration slope 1.00, and the Brier score 0.173, with bootstrap optimism-corrected values of −0.04, 0.92, and 0.183, respectively (Figure 3). The full coefficients of the combined model (*n* = 158; 63 events) are reported in Table 5.

**Figure 3:**
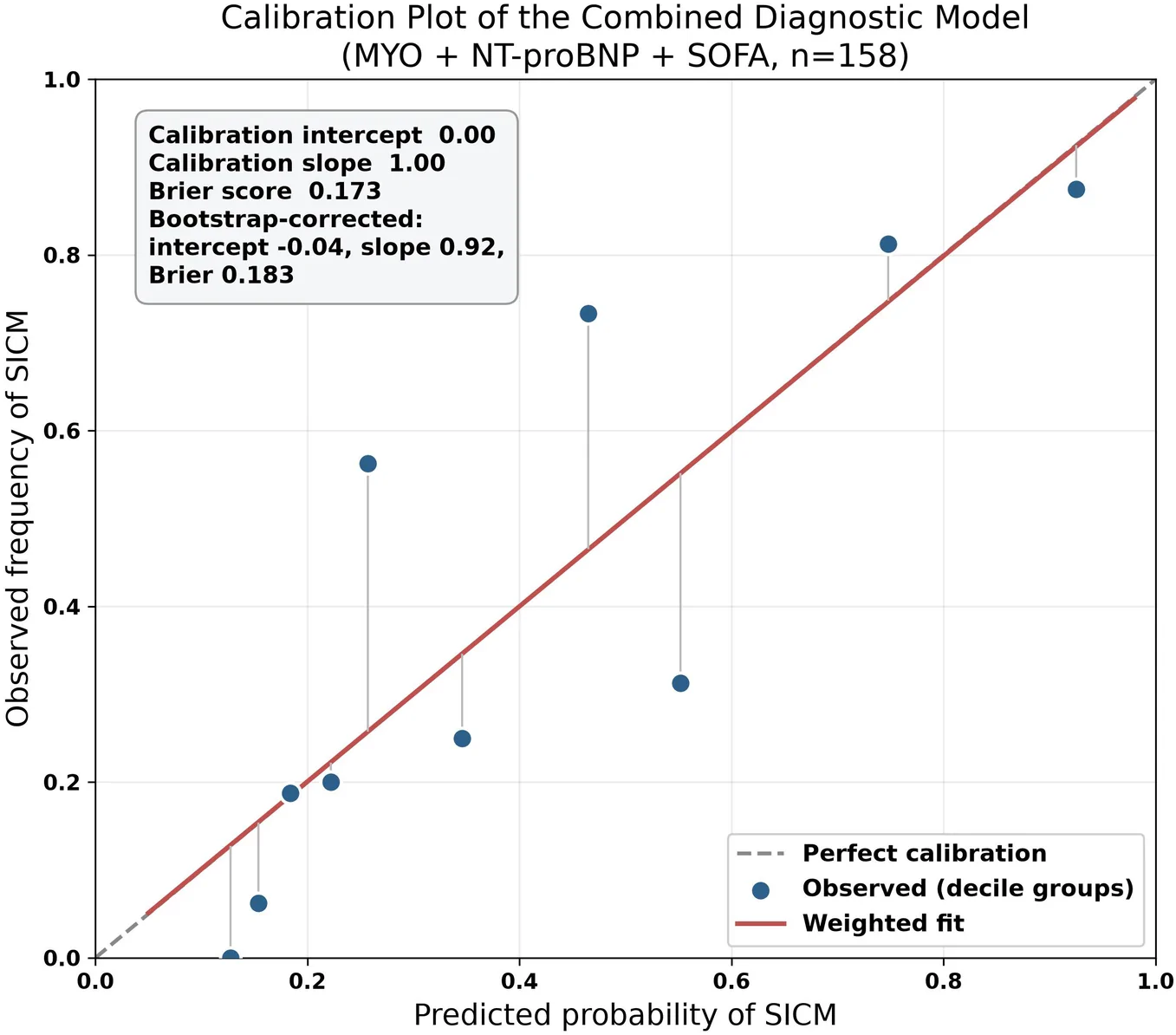
Calibration plot of the combined diagnostic model (MYO + NT-proBNP + SOFA) in the full cohort (*n* = 158). Predicted probabilities were grouped into ten quantiles; observed frequencies of SICM are plotted against mean predicted probabilities, with the dashed line indicating perfect calibration. Calibration intercept 0.00, calibration slope 1.00, Brier score 0.173; bootstrap optimism-corrected values −0.04, 0.92, and 0.183, respectively.

**Table 5:** Full coefficients of the combined diagnostic model (MYO + NT-proBNP + SOFA; *n* = 158, 63 events).

Variable	B	SE	P	OR	95% CI
Intercept	−2.428	0.459	<0.001	0.088	0.036–0.217
MYO (per 100 ng/mL)	0.070	0.025	0.006	1.073	1.021–1.127
NT-proBNP (per 1,000 pg/mL)	0.093	0.031	0.003	1.098	1.033–1.166
SOFA (per point)	0.172	0.061	0.005	1.188	1.053–1.339

B, regression coefficient; SE, standard error; OR, odds ratio; CI, confidence interval. The intercept corresponds to a patient with MYO = 0 ng/mL, NT-proBNP = 0 pg/mL, and SOFA = 0. *n* = 158 with 63 SICM events; complete data for all covariates.

### Decision curve analysis

Decision curve analysis suggested that the combined model provided a higher estimated net benefit than SOFA alone, the individual biomarkers (MYO; NT-proBNP), and the treat-all strategy across threshold probabilities of approximately 0.1–0.7 (Figure 4). This represents a theoretical net benefit under the assumptions of the decision curve framework; clinical effectiveness of model-guided decisions (e.g., expedited echocardiography for patients with a high model-based probability) requires prospective evaluation.

**Figure 4:**
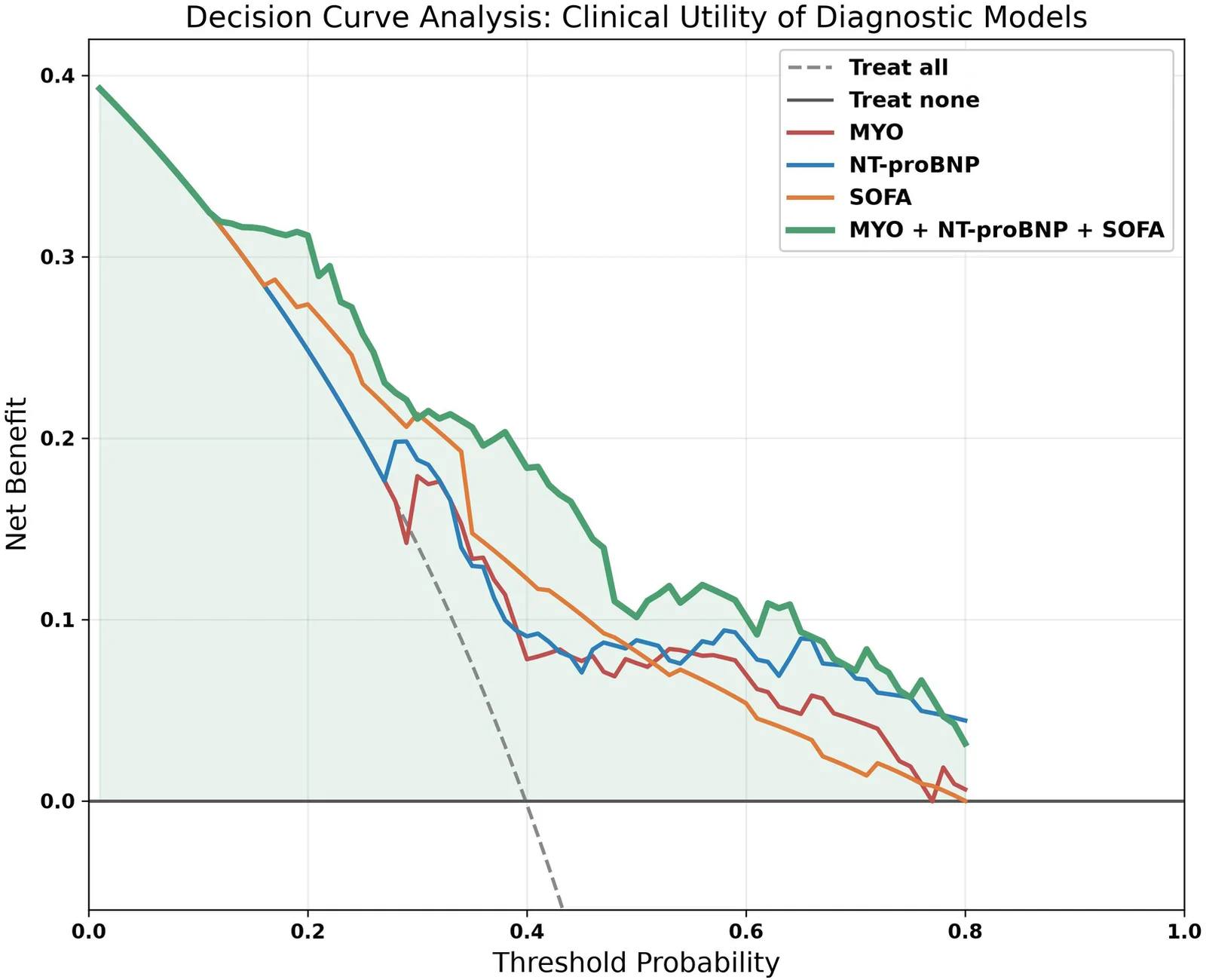
Decision curve analysis (DCA) evaluating the estimated clinical utility of the diagnostic models in the full cohort (*n* = 158), now including the SOFA-only model. The combined model (MYO + NT-proBNP + SOFA) demonstrated higher estimated net benefit than SOFA alone, the individual biomarkers (MYO; NT-proBNP), and the treat-all strategy across threshold probabilities of approximately 0.1-0.7. This represents a theoretical net benefit under decision-curve assumptions; clinical effectiveness requires prospective evaluation.

### Correlation analysis

MYO showed moderate positive correlations with the SOFA score (rho = 0.429), troponin I (rho = 0.420), and lactate (rho = 0.372), and correlated inversely with LVEF (rho = −0.272) and with eGFR (rho = −0.418). NT-proBNP correlated negatively with LVEF (rho = −0.374) and with eGFR (rho = −0.417). MYO and NT-proBNP were only weakly correlated with each other (rho = 0.285), suggesting that they capture complementary information.

## Discussion

Patients with SICM have poor prognosis, and the mechanisms underlying its occurrence are related to toxins, cytokines, nitric oxide, complement activation, apoptosis, and disordered energy metabolism (7–9). In this prospective cohort study of 158 patients with sepsis, admission MYO was independently associated with SICM after adjustment for demographic variables and renal function, and a combined model incorporating MYO, NT-proBNP, and the SOFA score achieved good discrimination (AUC 0.821; optimism-corrected AUC 0.812), significantly outperforming SOFA alone.

### Comparison with previous studies

Elevated MYO in sepsis and its association with disease severity have been reported previously (4). A prior prospective cohort evaluating MYO for septic cardiomyopathy reported an AUC of 0.545 (10), which is lower than our estimate of 0.704. This discrepancy may reflect differences in population characteristics (emergency department versus intensive care unit cohorts), sampling timing (admission versus later time points), and SICM diagnostic definitions. Our findings require external validation before broader generalizability can be claimed.

### Why myoglobin?

We selected MYO as the candidate biomarker for several reasons. First, MYO rises earlier than cardiac troponins after muscle injury, potentially enabling earlier detection of sepsis-associated myocardial dysfunction. Second, its short half-life permits dynamic monitoring. Third, because MYO is released from both cardiac and skeletal muscle in response to tissue hypoperfusion, we hypothesized it might serve as a composite marker of systemic hypoperfusion and myocardial stress. We acknowledge that cardiac troponin I is more specific for myocardial injury; in our cohort, TnI was also elevated in SICM patients and was associated with SICM in univariate analysis (Table 2). Future studies should directly compare the diagnostic performance of MYO against high-sensitivity cardiac troponin and against multimarker panels. Importantly, we emphasize that this study was not designed to establish MYO as a replacement for cardiac troponin. Because MYO lacks cardiac specificity, we envision its role as an early, inexpensive adjunctive screening marker used in combination with NT-proBNP and the SOFA score to identify patients who warrant expedited echocardiography, whereas troponin I remains the reference marker for cardiac-specific injury assessment. Whether MYO adds value beyond high-sensitivity troponin for SICM identification must be established in dedicated head-to-head comparisons.

### Combined model and clinical interpretation

NT-proBNP reflects cardiac wall stress and is an established prognostic marker in sepsis-associated myocardial dysfunction (11–13), consistent with the findings of Jeong et al. (14). The SOFA score captures the function of multiple organ systems and has been associated with SICM (15). The weak mutual correlation between MYO and NT-proBNP (rho = 0.285) suggests that they reflect complementary pathophysiological processes—tissue injury and hypoperfusion versus ventricular wall stress—providing a rationale for their combined use. To facilitate clinical application, the model can be applied to individual patients using the fitted equation. For example, a patient with a SOFA score of 8, MYO of 400 ng/mL, and NT-proBNP of 3,000 pg/mL has logit(P) = −2.428 + 0.172 × 8 + 0.0007 × 400 + 0.0001 × 3000 = −0.47, corresponding to a predicted probability of 0.38; using the Youden-derived probability cutoff of 0.383, this patient would be classified as likely to have SICM and prioritized for echocardiography. Where the full model cannot be computed, the single-marker cutoffs may serve as reference points: MYO 156.7 ng/mL, NT-proBNP 1,630 pg/mL, and SOFA 6. We caution that these thresholds were derived in a single cohort and require external validation before routine clinical use. The addition of MYO to the established SOFA score and NT-proBNP was associated with a statistically significant improvement in discrimination (likelihood ratio chi-square = 8.40, *P* = 0.002; IDI 0.041, 95% CI 0.001–0.134), and decision curve analysis indicated a net benefit across threshold probabilities of 0.1-0.7 (Figure 4). Although the absolute AUC increment was modest and the category-free NRI confidence interval included the null (0.383, 95% CI −0.030–0.806), MYO is inexpensive, routinely available in emergency departments, and measurable within hours of admission; we therefore consider the combined model a low-cost tool to prioritize echocardiography rather than a definitive diagnostic test, and its incremental value should be confirmed in larger, multicenter cohorts.

### Limitations

Our study has several important limitations. First, the generalizability of our findings is limited by the exclusion of patients with atrial fibrillation, prior cardiac disease, and other conditions, which was necessary to isolate sepsis-induced rather than pre-existing myocardial dysfunction; our results therefore apply to patients without significant baseline cardiac comorbidity. Second, MYO is not specific to myocardial injury and is also released from skeletal muscle in sepsis-related tissue hypoperfusion and inflammation (16–19). MYO correlated inversely with eGFR (rho = −0.42), and although MYO remained independently associated with SICM after adjustment for eGFR, renal dysfunction and skeletal muscle injury remain plausible partial explanations for elevated MYO. Future studies should incorporate muscle-specific markers (e.g., CK-MM isoforms) and dynamic renal assessment. Consistent with this concern, the association between MYO and SICM was attenuated and became non-significant after additional adjustment for CK-MB (see Results), underscoring that MYO should be interpreted as an adjunctive marker rather than a specific indicator of myocardial injury. Third, the definition of SICM relied on LVEF, which is load-dependent and may not reflect intrinsic myocardial contractility; low LVEF in septic shock has an uncertain association with mortality. We adopted the LVEF-based definition because it remains the most widely used criterion in the SICM literature (5, 6); speckle-tracking-derived global longitudinal strain may characterize SICM more sensitively (20–24). Fourth, baseline LVEF was not systematically available; the operational diagnosis relied on admission LVEF below 50% with clinical adjudication of reversibility. Follow-up echocardiography was completed in 45 of the 63 patients (71.4%) with SICM and confirmed recovery in all of them; in the remaining 18 patients (six deaths, seven early discharges or transfers, and five without repeat imaging) reversibility could not be documented, and some degree of misclassification cannot be excluded. Fifth, patients who died within 72 h before echocardiography could be performed were excluded, which may introduce selection bias by removing the most severely ill patients. Sixth, biomarkers were measured at admission, concurrently with diagnostic imaging; therefore, our findings support early diagnosis at presentation rather than true prospective prediction of incident SICM. Seventh, this was a single-center study, and although bootstrap internal validation suggested minimal overfitting (optimism-corrected AUC 0.812), external validation in an independent multicenter cohort is required. Finally, the net benefit suggested by decision curve analysis is theoretical; whether model-guided management improves patient outcomes must be tested in prospective implementation studies.

### Future directions

Future research should expand sample sizes and adopt multicenter designs to validate our findings. Incorporating patient outcomes and advanced imaging—particularly speckle-tracking echocardiography and global longitudinal strain—may improve the accuracy of SICM diagnosis and prognostic assessment. Combining MYO with other candidate biomarkers and clinical scores warrants further investigation.

## Data Availability

The original contributions presented in the study are included in the article/Supplementary Material, further inquiries can be directed to the corresponding author.
